# The effect of Tai Chi on elderly depression: a systematic review and meta-analysis of randomized controlled trials

**DOI:** 10.3389/fpsyg.2024.1489384

**Published:** 2024-11-29

**Authors:** Fengya Zhu, Yuan Wang, Shao Yin, Junqian Liu, Yue Zhong, Liuying Li

**Affiliations:** ^1^Zigong First People’s Hospital, Zigong, China; ^2^Acupuncture and Tuina School, Chengdu University of Traditional Chinese Medicine, Chengdu, China; ^3^Hospital of Chengdu University of Traditional Chinese Medicine, Chengdu, China

**Keywords:** elderly, depression, Tai Chi, quality of life, meta-analysis

## Abstract

**Objective:**

This systematic review and meta-analysis assess the impact of Tai Chi on emotional well-being and quality of life in elderly individuals with depression.

**Methods:**

Searching six databases until October 20, 2024, including Embase, PubMed, Cochrane Library, Web of Science, SinoMed, and CNKI, for randomized controlled trials (RCTs). Participants were aged ≥60 and diagnosed with depression. Tai Chi served as the main intervention in the treatment group, with the control group receiving no exercise, or only health education. The primary outcomes focused on the improvement of depressive symptoms and quality of life. Data synthesis and meta-analysis were performed using Stata 15.1 software. The protocol of this study was registered on PROSPERO (ID: CRD42023479305).

**Results:**

Tai Chi, as the main intervention, significantly improved depressive symptoms as measured by the Geriatric Depression Scale (WMD = −5.99, 95% CI: −10.80 to −1.19, *p* = 0.015) compared to no exercise or health education. Subgroup analysis favored a six-month duration of Tai Chi, showing even greater benefits (WMD = −9.79, 95% CI: −13.90 to −5.69, *p* < 0.001). However, Tai Chi did not demonstrate a significant advantage in improving participants’ scores on the Hamilton Depression Scale (WMD = −1.04, 95% CI: −3.84 to 1.76, *p* = 0.468).

**Conclusion:**

Our results indicate that Tai Chi can significantly improve depressive symptoms and quality of life in elderly individuals with depression. As a daily exercise and mind–body therapy to alleviate the mood of the elderly, it is necessary to conduct more large-sample RCTs. Further research on the details of Tai Chi, such as movements, frequency, duration, and exercise periods, is essential for a dose–response relationship, contributing to the standardized promotion of Tai Chi.

## 1 Introduction

The global elderly population is rapidly increasing. In 2019, the population over 60 years old has already exceeded one billion, and it is expected to reach two billion by 2050 (Economic UNDo, Affairs S, 2020). There is a growing emphasis on healthy aging, often referred to as the “ultimate preventive medicine” (Kaeberlein et al., 2015). However, in the current social context, a significant number of elderly individuals find themselves living alone or in nursing homes, contributing to heightened feelings of loneliness. While loneliness is recognized as a significant factor in the development of depression, it is important to acknowledge that the increase in depression rates among the elderly is multifaceted. Various factors, including social isolation, economic hardships, and the impact of the pandemic, also play crucial roles. Consequently, this has resulted in high rates of elderly depression in both developed and developing countries (Zhang et al., 2023; Zhao et al., 2018; Domènech-Abella et al., 2017). According to statistics, the prevalence of community-based elderly depression is 27.5% in the United States (Laborde-Lahoz et al., 2015) and 20% in China (Tang et al., 2021), with an overall prevalence of 36.8% among elderly residents in nursing homes (Tang et al., 2022). Among the elderly, depression is considered the second most common mental health disorder (Panza et al., 2010), and is also one of the most common neuro-psychiatric precursors to dementia (Roberto et al., 2021). Depression results in a significant decline in an individual’s quality of life and has profound impacts on disability (Friedrich, 2017), executive function (Lockwood et al., 2002), falls (Iaboni and Flint, 2013), cardiovascular diseases and strokes (Lépine and Briley, 2011), as well as deaths caused by various reasons (Murphy et al., 1988).

Age is considered an important variable associated with disease deterioration (Licht-Strunk et al., 2007). Evidence indicates that, compared to younger individuals, elderly depression has a higher risk of recurrence (Licht-Strunk et al., 2009), and a lower rate of response to medications. Elderly depression is more susceptible to the impact of medication side effects, has a longer duration and poorer prognosis (Knöchel et al., 2015), and is associated with an increased risk of mortality (Rovner et al., 1991), disability(Lenze et al., 2001), and medical expenses (Katon et al., 2003). Therefore, there is a need for supplementary treatment strategies to enhance the therapeutic outcomes of elderly depression, and exercise appears to be a non-pharmacological treatment strategy particularly suitable for elderly depression (Bridle et al., 2012).

Over the past few decades, the evidence supporting the health benefits of Tai Chi has continually increased, especially regarding its significance for elderly populations. For instance, Tai Chi has been shown to prevent falls and improve balance (Huang et al., 2022; Chen et al., 2023), which is essential for preserving independence among older adults. Additionally, it reduces the occurrence of falls in Parkinson’s disease patients (Winser et al., 2018), aids in stroke recovery(Zhao et al., 2022), and lowers hypertension (Wu et al., 2021), all of which contribute to enhanced physical health. Furthermore, Tai Chi is associated with the prevention of cardiovascular diseases (Lee et al., 2007), and improvements in cognitive function (Chen et al., 2023; Sungkarat et al., 2018). It also assists in the treatment of metabolic syndrome (Chau et al., 2021), reducing waist circumference in centrally obese patients (Siu et al., 2021), and alleviating knee osteoarthritis pain (Liu et al., 2019), which highlights its versatility as a holistic exercise. Given that Tai Chi can be practiced nearly anywhere and is usually performed at moderate intensity (Cheng, 1999), while presenting a low risk of serious adverse events (Yang et al., 2022), it is considered a safe exercise.

Growing scientific evidence suggests that Tai Chi can improve the mental and physical health of the elderly, cognitive and motor learning habits (Solianik et al., 2021; Nickles et al., 2010), enhance sleep quality (Irwin et al., 2008; Wang et al., 2023; Siu et al., 2021), and alleviate depression (Liu et al., 2018) thus improving the overall quality of life (Sani et al., 2023). Additionally, studies indicate positive effects of Tai Chi on elderly depression patients, including emotions (Liao et al., 2018; Lavretsky et al., 2022; Siddarth et al., 2023), blood glucose and blood pressure (Wang et al., 2022), health function, and cognitive performance (Lavretsky et al., 2011), as well as overall quality of life (Liao et al., 2019). Currently, only one meta-analysis has assessed the efficacy of Tai Chi for depression in middle-aged and elderly individuals. However, we found that among the included 12 RCTs, the participants encompassed various groups, including middle-aged and elderly individuals with depression, as well as healthy elderly individuals in the community or nursing homes (Zeng et al., 2023). Therefore, the purpose of this study is to clarify the impact of Tai Chi on elderly depression through a systematic review and meta-analysis.

## 2 Methods

This study has been registered on the PROSPERO (ID: CRD42023479305) https://www.crd.york.ac.uk/PROSPERO/display_record.php?RecordID=479305.

### 2.1 Eligibility criteria

Inclusion criteria: (1) The study design must be a randomized controlled trial(RCT). (2) Participants should be elderly depression patients aged ≥60, with clear diagnostic criteria. (3) Tai Chi should be the primary intervention (with no restrictions imposed on the style of Tai Chi, training duration, length of each session, or frequency of training), and the control group should receive interventions other than Tai Chi, such as Western medicine, health education, or no intervention. (4) The RCT should assess at least one depression symptom-related score.

Exclusion criteria: (1) Age < 60 years. (2) Elderly subjects without depression. (3) Unclear diagnostic criteria. (4) Studies of other types, such as non-RCTs, observational studies, case–control studies. (5) Direct comparisons of different types, frequencies, or durations of Tai Chi exercises. (6) Lack of any specific outcome indicators. (7) Secondary analysis of data.

### 2.2 Search strategy

We searched six databases, including Embase, PubMed, Cochrane Library, Web of Science, SinoMed, and CNKI. The search was conducted up to October 20, 2024 The search terms primarily included Tai Chi, depression, elderly, and RCT. Additionally, we manually searched relevant reference lists. Grey literature and data results on research registration platforms are not considered within the scope as we did not have access permission. Detailed search strategies and exclusion criteria are provided in Supplementary material.

### 2.3 Study selection

Two reviewers (SY and FYZ) independently conducted searches and screenings of potential literature based on a pre-established search strategy. Relevant entries were imported into Endnote 21 to eliminate duplicate articles. Preliminary screening was then conducted by reading titles and abstracts, followed by a thorough examination of the full text to determine the final eligible studies. Any disagreements were resolved through discussion. If consensus could not be reached, the final decision was made by a third reviewer (LYL).

### 2.4 Data extraction

Following a pre-established extraction plan, two reviewers independently extracted data from eligible literature. The extraction content included author and publication year, diagnostic criteria, participant information, sample size, intervention measures and details, outcomes, etc. After independent extraction, a cross-check was performed. Any discrepancies were resolved by the third reviewer (LYL) for the final decision.

### 2.5 Assessment of the risk of bias

Two reviewers (SY and FYZ) independently used the Cochrane RoB 2 tool to assess the risk of bias for each study. This evaluation included five aspects: randomization process, deviations from intended interventions, missing outcome data, measurement of the outcome, and selection of the reported result. The final overall bias risk for each study was determined as low risk, some concerns, or high risk. Any discrepancies were resolved by the third reviewer (LYL) for the final decision.

### 2.6 Data synthesis and statistical analysis

Statistical analysis was conducted using Stata 15.1 software. Continuous data were analyzed using the weighted mean difference (WMD) and 95% confidence interval (CI). For all analysis results, *p* < 0.05 was considered statistically significant. If there was high heterogeneity (I^2^ ≥ 50% or *p* < 0.05), a random-effects model was used; otherwise, a fixed-effects model was employed. Subgroup analysis was conducted based on the total exercise duration. Sensitivity analysis was performed to assess the stability of the test results, and Begg’s and Egger’s tests were used to evaluate publication bias.

## 3 Result

### 3.1 Results on literature search and selection

**Figure 1:**
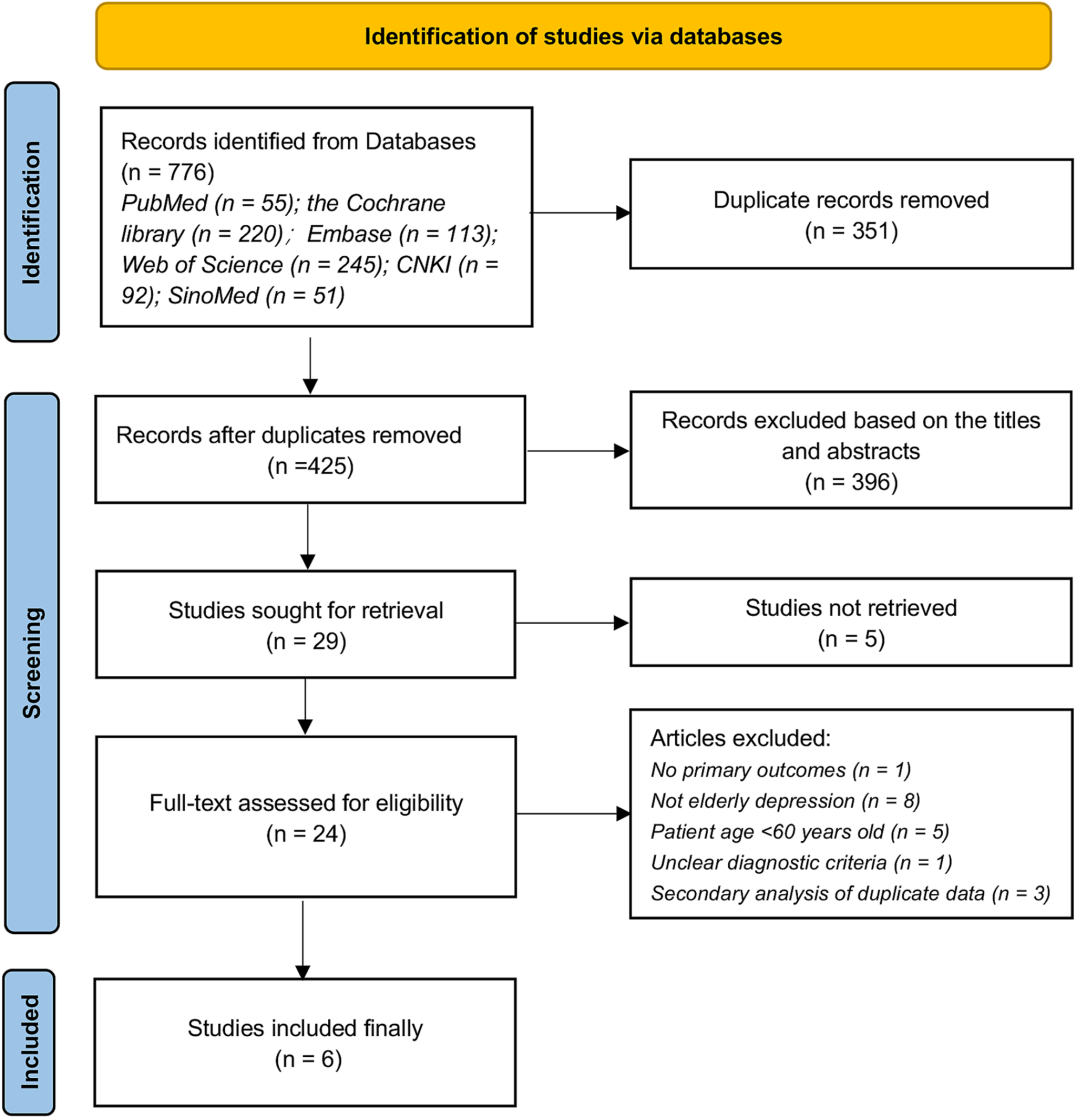
Literature search flowchart.

We retrieved a total of 743 relevant articles from 6 databases, excluding 351 duplicates. Following a comprehensive evaluation based on titles, abstracts, and full-text reading, 6 qualified studies were ultimately included for analysis. The detailed flowchart is presented in Figure 1. The exclusion list and reasons for exclusion during the ‘Full-text assessed for eligibility’ stage are provided in Supplementary material.

### 3.2 Characteristics of included study

This study included a total of 6 RCTs, comprising 495 patients, with 249 in the treatment group and 246 in the control group. The studies were conducted between 2004 and 2022 in China, the USA, and Malaysia. Participants were elderly individuals with depression, diagnosed using either the Diagnostic and Statistical Manual of Mental Disorders, Fourth Edition, or Geriatric Depression Scale (GDS) with a score ≥ 10. All patients were aged 60 and above. The Tai Chi exercise frequency ranged from 1 to 5 times per week, with each session lasting between 45 and 120 min. The total duration varied from 10 weeks to 6 months. All studies assessed depression-related outcomes, primarily using GDS, with no studies reporting adverse events. Detailed characteristics of the literature are presented in Table 1.

**Table 1:** Characteristics of the literature.

Included studies	Location	Diagnostic criteria	Sample size (I/C)	Age [mean (SD)] (I/C)	Sex (male/female) (I, C)	Intervention	Comparison	Duration	Outcomes	Adverse events (I/C)
Chou et al. (2004)	China	DSM-IV	7/7	72.6 (4.2)	7/7	TC (45 min three times a week)	Waiting list	3 m	CES-D	NR
Guixiang (2012)	China	GDS > 10 score	35/33	> 60	31/37	TC (60 min five times a week)	No treatment	6 m	GDS	NR
Lavretsky et al. (2011)	USA	DSM-IV	33/35	69.1 (7.0)/72.0 (7.4)	10/23, 13/22	TC (120 min once a week) + Escitalopram (20 mg/once a day)	Health education (2 h per week) + Escitalopram (20 mg/once a day)	10 w	HAM-D, SF-36	NR
Liao et al. (2018)	Malaysia	GDS > 10 score	55/52	71.84 (7.297)/71.75 (8.201)	19/36, 22/30	TC (50 min three times a week)	Health education (once a month)	3 m	GDS	NR
Liu et al. (2018)	China	GDS ≧10 score	30/30	60.90 (4.28)/61.72 (3.54)	14/16, 14/16	TC (60 min three times a week)	No treatment	6 m	GDS	NR
Lavretsky et al. (2022)	USA	DSM-IV/DSM-5	89/89	69.2 (6.9)/69.4 (6.2)	62/27, 67/22	TC (60 min once a week)	Health education (60 min once a week)	3 m	GDS, HAM-D, SF-36	NR

DSM, Diagnostic and Statistical Manual of Mental Disorders; TC, Tai Chi; I, intervention group; C, comparison group; NR, not reported; w, week; m, month; CES-D, Center for Epidemiological Studies Depression Scale; GDS, Geriatric Depression Scale; HAM-D,Hamilton Depression Scale; SF-36, Short Form 36 Health Survey.

### 3.3 Risk of bias

**Figure 2:**
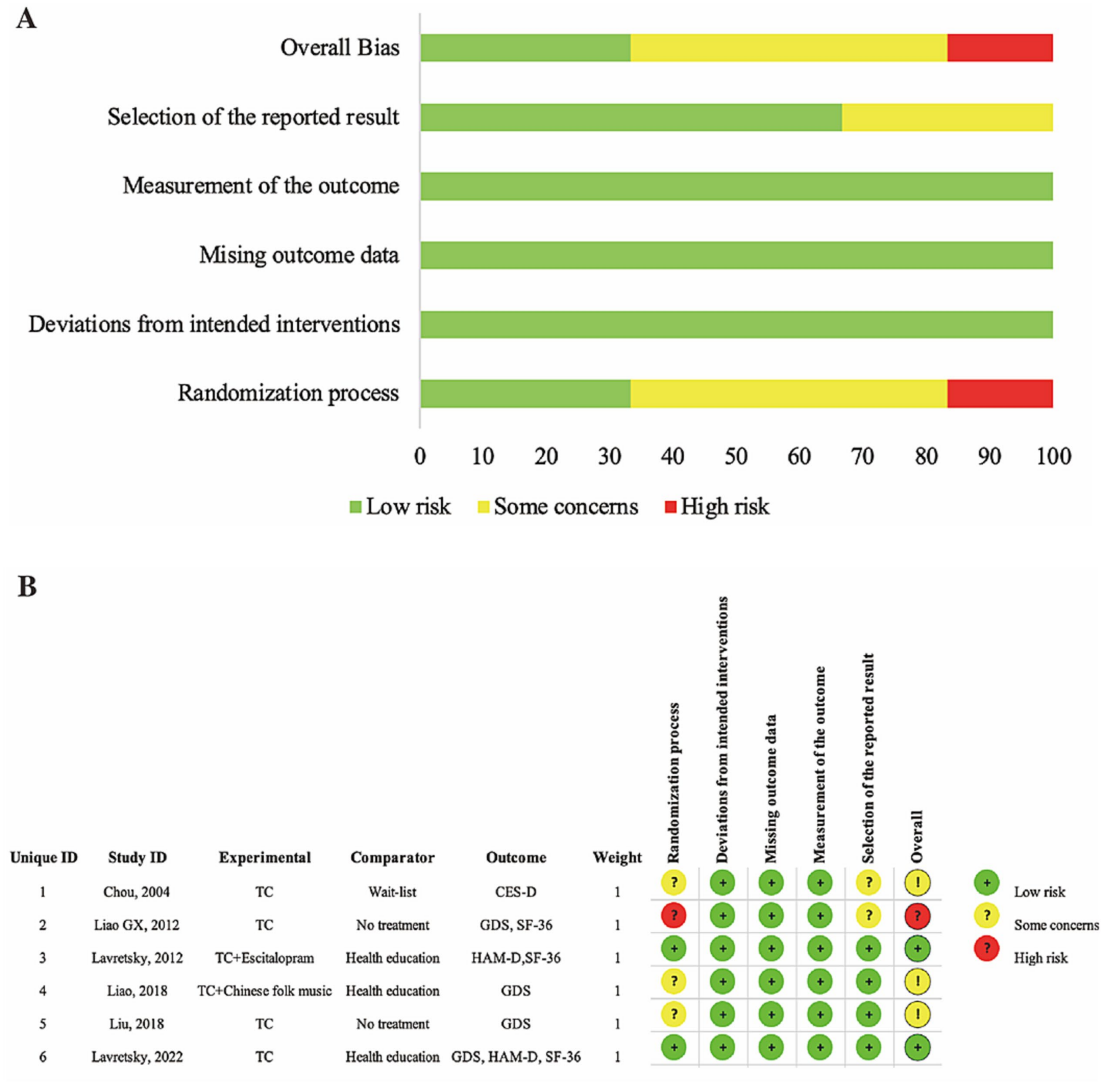
**(A)** Risk of bias item presented as percentages across all included RCTs. **(B)** Risk of bias item for included RCTs.

We conducted a bias risk assessment for the 7 RCTs using Cochrane RoB 2. Three studies were rated as “some concerns” due to the absence of mentioning allocation concealment or the presence of bias in other aspects. One study was rated as “high risk” because it did not specify random methods and allocation concealment, and bias was present in other aspects. The remaining three studies were classified as “low risk,” and specific details of bias risks are shown in Figure 2.

### 3.4 Results of the meta-analysis

**Figure 3:**
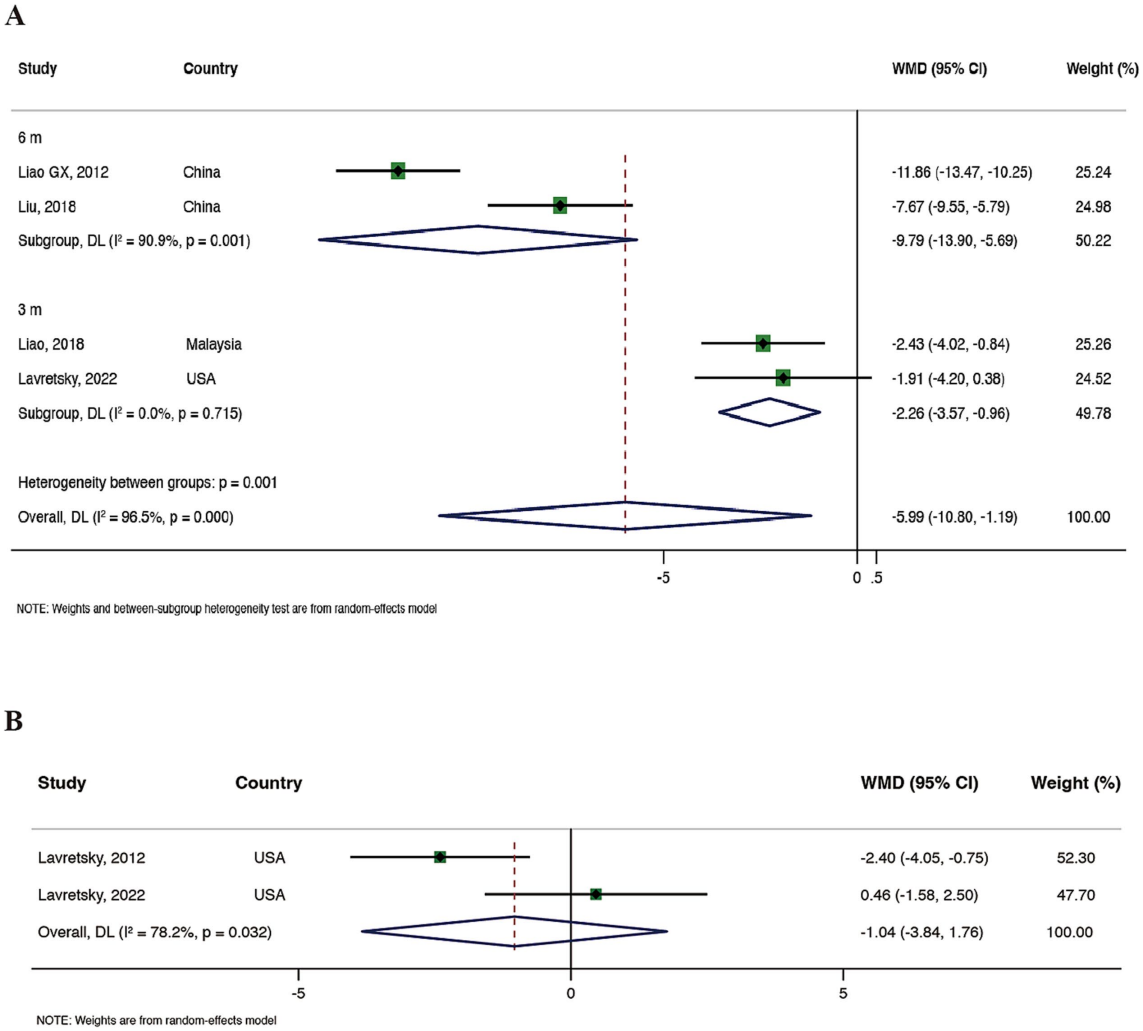
**(A)** Meta-analysis of GDS. **(B)** Meta-analysis of HAMD.

Four RCTs (Liu et al., 2018; Liao et al., 2018; Lavretsky et al., 2022; Guixiang, 2012) reported on GDS. Meta-analysis results showed that compared to the control group, Tai Chi demonstrated a significant advantage in improving patients’ depressive symptoms (WMD = −5.99, 95% CI: −10.80 to −1.19, and *p* = 0.015), but with high heterogeneity (*I*^2^ = 96.5%, *p* = 0.000). We conducted a subgroup analysis based on the total duration of Tai Chi exercise. The results indicated that a 6-month Tai Chi exercise better improved patients’ depressive symptoms (WMD = −9.79, 95% CI: −13.90 to −5.69, and *p* < 0.001), although with high heterogeneity (*I*^2^ = 90.0%, *p* = 0.001). Meta-analysis results are detailed in Figure 3A. Finally, we conducted a sensitivity analysis on this outcome, indicating the stability of the results (see Supplementary material).

Two RCTs (Lavretsky et al., 2022; Lavretsky et al., 2011) assessed the Hamilton Depression Scale (HAMD). Meta-analysis results showed that compared to health education, Tai Chi did not exhibit a significant advantage in improving participants’ depressive symptoms (WMD = −1.04, 95% CI: −3.84 to 1.76, and *p* = 0.468) with higher heterogeneity (*I*^2^ = 78.2%, *p* = 0.032), see Figure 3B for details. Sensitivity analysis showed stable results (see Supplementary material).

### 3.5 Summary of the outcomes

One RCT (Chou et al., 2004) assessed the Center for Epidemiological Studies Depression Scale (CES-D). The results indicated that after 3 months of Tai Chi intervention, the intervention group showed a significant improvement in depressive symptoms compared to the control group (*p* < 0.05). Two RCTs (Lavretsky et al., 2022; Lavretsky et al., 2011) evaluated the SF-36 General Health, SF-36 physical functioning and SF-36 role emotional. Both of their results suggested that, compared to health education, Tai Chi significantly improved the overall health of elderly patients with depression (10 weeks, *p* = 0.02; 6 months, *p* = 0.003).

## 4 Discussion

This study focuses on whether Tai Chi is beneficial for depressive symptoms in elderly patients with depression. Through rigorous selection criteria, we ultimately analyzed 6 RCTs. Overall, compared to no intervention or only health education, long-term Tai Chi exercise can significantly alleviate the depressive mood of elderly individuals with depression.

Tai Chi represents a broad philosophy and theoretical concept, describing the spontaneous dynamic equilibrium state in the interactions of the natural world (i.e., the universe). Originating in China, Tai Chi is a traditional mind–body exercise characterized by gentle movements that promote relaxation and well-being. It requires focused concentration, stable breathing, and relaxation of the body during slow movements (Birdee et al., 2009), with the added benefits of slow-paced breathing (Hoffmann et al., 2019; Laborde et al., 2022; Laborde et al., 2022; Laborde et al., 2017; Laborde et al., 2019; Laborde et al., 2019; You et al., 2021). Maintaining the balance of Yin and Yang in the body is crucial for health, and practicing Tai Chi helps promote the flow of Qi within the body, achieving a state of Yin-Yang balance (Elinwood, 2002).

In terms of overall mental and physical health, Tai Chi serves as a beneficial mind–body exercise that supports healthy aging among elderly residents. This practice encourages individuals to gather together, expanding their social circles and fostering increased communication (Lee and Chu, 2023). Tai Chi not only emphasizes physical movement but also highlights mindfulness and inner balance. Its slow, graceful movements prompt elderly individuals to focus on their bodily sensations, which can alleviate anxiety and stress, thereby providing a valuable option for this population (Xianjian and Datao, 2021; Cheng et al., 2021; Cheng et al., 2023).

However, there is still a lack of standardized research on Tai Chi, with variations in the frequency, duration, and total exercise time across studies. Due to the limited number of articles, we conducted an analysis based solely on the total duration and found that a 6-month exercise period is more effective in improving symptoms of elderly depression. However, the dose-effect relationship between the intensity of Tai Chi exercise and its effects still requires further investigation.

Due to the stringent criteria we employed, limiting all participants to those aged 60 and above with a clear diagnosis of depression, the final number of included RCTs for analysis was relatively small, making it challenging for us to explain the high heterogeneity. Secondly, the small sample sizes of the included trials may impact the risk of bias in the results. This study primarily focuses on the impact of Tai Chi on the depression and quality of life of elderly depression patients, with other aspects of results not considered within the scope. To further clarify the positive effects of Tai Chi on the mental and physical health of elderly depression patients, future research should involve larger sample sizes. Additionally, standardizing details such as Tai Chi movements, frequency, duration, and exercise periods is crucial to facilitate the broader promotion of Tai Chi.

## 5 Conclusion

Our results indicate that compared to no exercise or mere health education, Tai Chi can significantly improve depressive symptoms and quality of life in elderly depression patients. Tai Chi, as a daily exercise and mind–body therapy to alleviate the mood of the elderly, deserves further affirmation of the accuracy and stability of research results.

## Data Availability

The original contributions presented in the study are included in the article/Supplementary material, further inquiries can be directed to the corresponding author.
