# Supplementary material for: The effect of Tai Chi on elderly depression: a systematic review and meta-analysis of randomized controlled trials
Source: Front Psychol. 2024 Nov 29;15:1489384. doi: 10.3389/fpsyg.2024.1489384 (PMC11637854; doi:10.3389/fpsyg.2024.1489384)
Supplement: Supplementary file 1 [file Data_Sheet_1.PDF]

# Supplementary material

| Database | Search strategy | Results |
|---|---|---|
| PubMed | (((((((((Tai Chi[MeSH Terms]) OR (Taichi[Text Word])) OR (Tai Chi Chuan[Text Word])) OR (Tai Ji[Text Word])) OR (Taiji[Text Word])) OR (Taijiquan[Text Word])) OR (T'ai Chi[Text Word])) AND ((((((((Aged[MeSH Terms]) OR (elderly[Text Word])) OR (Old[Text Word])) OR (old people[Text Word])) OR (older adults[Text Word])) OR (geriatric[Text Word])) OR (senior citizens[Text Word])) OR (late life[Text Word]))) AND (((((depression[MeSH Terms]) OR (depressive[Text Word])) OR (depressed[Text Word])) OR (depressive symptom[Text Word])) OR (depressive disorder[Text Word]))) AND ((((randomized controlled trial[Publication Type]) OR (clinical trial[Publication Type])) OR (trial[Text Word])) OR (randomized[Text Word])) | 55 |
| Embase | #1 taichi:ti,ab,kw OR 'tai chi chuan':ti,ab,kw OR 'tai ji':ti,ab,kw OR taiji:ti,ab,kw OR taijiquan:ti,ab,kw OR 'tai chi':ti,ab,kw<br><br>#2 aged:ti,ab,kw OR elderly:ti,ab,kw OR old:ti,ab,kw OR 'old people':ti,ab,kw OR 'older adults':ti,ab,kw OR geriatric:ti,ab,kw OR 'senior citizens':ti,ab,kw OR 'late life':ti,ab,kw<br><br>#3 depression:ti,ab,kw OR depressive:ti,ab,kw OR depressed:ti,ab,kw OR 'depressive symptom':ti,ab,kw<br><br>#4 'randomized controlled trial':it OR 'clinical trial':it OR trial OR randomized<br><br>#5 #1 AND #2 AND #3 AND #4 | 113 |
| The Cochrane library | #1 (Tai Chi):ti,ab,kw OR (Taichi):ti,ab,kw OR (Tai Chi Chuan):ti,ab,kw OR (Tai Ji):ti,ab,kw OR (Taiji):ti,ab,kw OR (Taijiquan):ti,ab,kw OR (T'ai Chi):ti,ab,kw<br><br>#2 (Aged):ti,ab,kw OR (elderly):ti,ab,kw OR (Old):ti,ab,kw OR (old people):ti,ab,kw OR (older adults):ti,ab,kw (Word variations have been searched) OR (geriatric):ti,ab,kw OR (senior citizens):ti,ab,kw OR (late life):ti,ab,kw)<br><br>#3 (depression):ti,ab,kw OR (depressive):ti,ab,kw OR (depressed):ti,ab,kw OR (depressive symptom):ti,ab,kw OR | 220 |

| | | |
|---|---|---|
| | (depressive disorder):ti,ab,kw<br>#4 (randomized controlled trial):ti,ab,kw OR (clinical trial):ti,ab,kw OR (trial):ti,ab,kw OR (Randomized):ti,ab,kw<br>#5 #1 and #2 and #3 and #4 | |
| Web of Science | (((TS=(Tai Chi OR Taichi OR Tai Chi Chuan OR Tai Ji OR Taiji OR Taijiquan OR T'ai Chi)) AND TS=(Aged OR elderly OR Old OR old people OR older adults OR geriatric OR senior citizens OR late life)) AND TS=(depression OR depressive OR depressed OR depressive symptom OR depressive disorder)) AND TS=(randomized controlled trial OR clinical trial OR trial OR Randomized) | 245 |
| CNKI | SU %=( '太极' + '太极拳') AND SU %= ( '抑郁' + '抑郁症' + '忧郁' ) AND SU %=( '老年' + '老龄化') | 92 |
| SinoMed | ("老年"[常用字段:智能] OR "老龄化"[常用字段:智能]) AND ("抑郁"[常用字段:智能] OR "抑郁症"[常用字段:智能] OR "忧郁"[常用字段:智能]) AND ("太极"[常用字段:智能] OR "太极拳"[常用字段:智能]) | 51 |

| **Exclusion list** | |
|---|---|
| **Reference** | **Reason** |
| Lin Youbiao, Zhang Shunjiao, Ye Zhanhong, et al. Effect of exercise prescription intervention on physical and mental health of elderly women [J]. Chinese Journal of Gerontology,2009,29(03):350-352. (in Chinese) | Not elderly depression |
| Zheng Jin, Wang Li, Zhou Invention, et al. Effect of simplified Taijiquan exercise on improving the quality of life of elderly women [J]. Journal of Xinjiang Medical University,2021,44(02):234-237. (in Chinese) | Not elderly depression |

| | |
|---|---|
| Li Yaqi. A randomized controlled experimental study on the influence of eight-style Taijiquan exercise on mental health level of elderly people in nursing homes [D]. Shandong University of Physical Education,2021. (in Chinese) | Not elderly depression |
| Ge Yujie. The intervention effect of simple style Taijiquan training on the debilitation level and exercise ability of the elderly in the pre-debilitation period [D]. North China University of Science and Technology,2021. (in Chinese) | Not elderly depression |
| Mo Xineng, Wang Bin. Influence of fitness Qigong and Tai Chi health stick exercise on mood state and mental health of elderly women [J]. Chinese Journal of Gerontology,2016,36(21):5401-5403. (in Chinese) | Not elderly depression |
| Liu Qiang. Effects of 16-week Taijiquan exercise and 8-week suspension on mental health of elderly women [J]. Journal of Shandong University of Physical Education,2016,32(06):99-103. (in Chinese) | Not elderly depression |
| Solianik R, Mickevičienė D, Žlibinaitė L, et al. Tai chi improves psychoemotional state, cognition, and motor learning in older adults during the COVID-19 pandemic. Exp Gerontol. 2021 Jul 15; 150:111363. | Not elderly depression |
| Xie Huihui. Effect of Taijiquan on depression and heart rate variability in middle-aged and elderly people.2011. Shanghai Institute of Physical Education,MA thesis. | Patient age <60 years old |
| Lin Haidi, Yu Jin, Zhang Feng. Effect of Taijiquan on emotion of elderly patients with depression during recovery period [J]. Chinese Community Physician,2018,34(35):168+170. (in Chinese) | Patient age <60 years old |
| Zhao Guizeng, Cheng Ruihuan, Jie Chongchong, et al. Effect of Taijiquan on mild depression in middle-aged and elderly people [J]. | Patient age <60 years old |

| | |
|---|---|
| Chinese Journal of Convalescent Medicine,2015,24(05):452-454. (in Chinese) | |
| Yeung AS, Feng R, Kim DJH, et al. A Pilot, Randomized Controlled Study of Tai Chi With Passive and Active Controls in the Treatment of Depressed Chinese Americans. J Clin Psychiatry. 2017 May;78(5): e522-e528. | Patient age <60 years old |
| Yeung A, Lepoutre V, Wayne P, et al. Tai chi treatment for depression in Chinese Americans: a pilot study. Am J Phys Med Rehabil. 2012 Oct;91(10):863-70. | Patient age <60 years old |
| Cho KL. Effect of Tai Chi on depressive symptoms amongst Chinese older patients with major depression: the role of social support. Med Sport Sci. 2008; 52:146-154. | Secondary analysis of duplicate data |
| Zhou Jie, Jia Long, Guan Tieyu, et al. Effect of Taijiquan on depression and anxiety in elderly women [J]. Sports & Sports,2012(04):17. (in Chinese) | Unclear diagnostic criteria |
| Wang, Yan et al. "Comparison of the effects of Tai Chi and general aerobic exercise on weight, blood pressure and glycemic control among older persons with depressive symptoms: a randomized trial." BMC geriatrics vol. 22,1 401. 7 May. 2022, doi:10.1186/s12877-022-03084-6 | Secondary analysis of duplicate data |
| Liao SJ, Chong MC, Tan MP, Chua YP. Tai Chi with music improves quality of life among community-dwelling older persons with mild to moderate depressive symptoms: A cluster randomized controlled trial. Geriatr Nurs. 2019;40(2):154-159. doi: 10.1016/j.gerinurse.2018.08.001 | Secondary analysis of duplicate data |
| Siddarth P, Abikenari M, Grzenda A, et al. Inflammatory Markers of Geriatric Depression Response to Tai Chi or Health Education Adjunct Interventions. Am J Geriatr Psychiatry.2023;31(1):22-32. doi: 10.1016/j.jagp.2022.08.004 | No primary outcomes |

| | |
|---|---|
| Chang S, Cheng L, Liu H. Effects of three-duration Tai-Chi exercises on depression and sleep quality in older women. Eur Geriatr Med. 2024 Aug;15(4):1141-1148. doi: 10.1007/s41999-024-00981-4. Epub 2024 May 1. PMID: 38693298. | Not elderly depression |

**Sensitivity analysis**

GDS

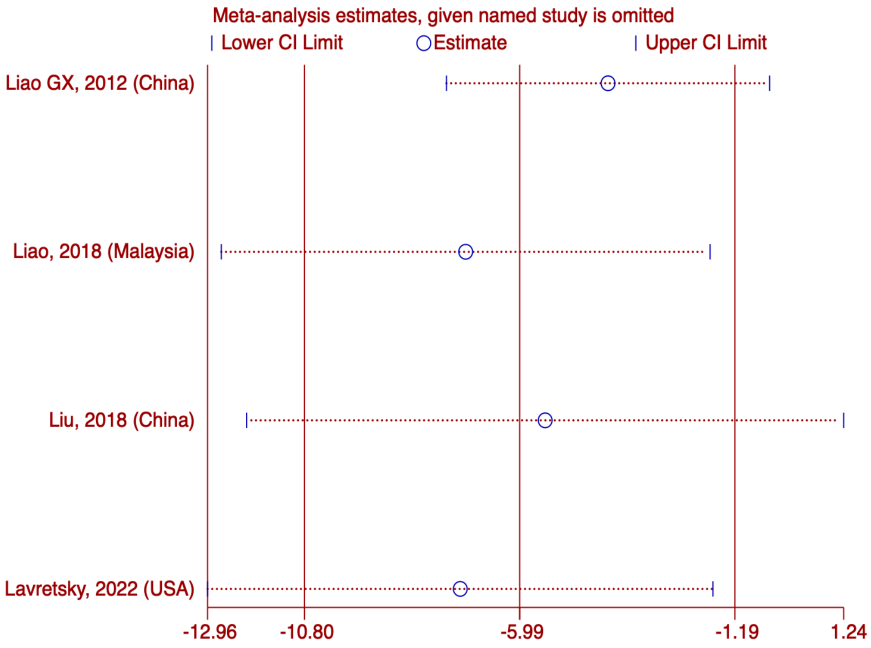


HAMD

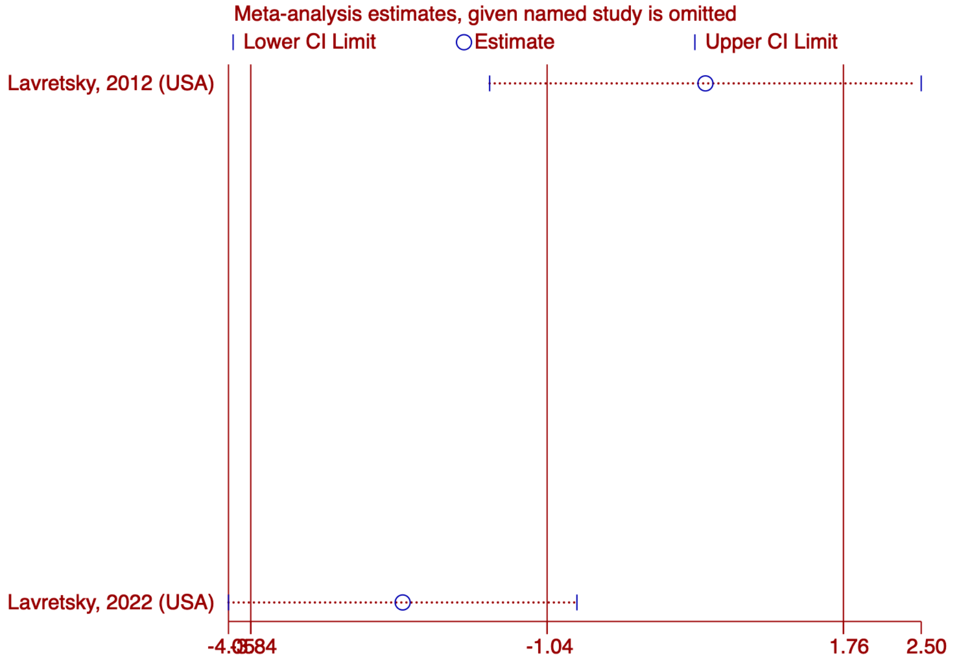
Meta-analysis estimates, given named study is omitted
Lower CI Limit
Estimate
Upper CI Limit
Lavretsky, 2012 (USA)
Lavretsky, 2022 (USA)
-1.04
1.76
2.50
